# Deep learning-based image quality improvement of ^18^F-fluorodeoxyglucose positron emission tomography: a retrospective observational study

**DOI:** 10.1186/s40658-021-00377-4

**Published:** 2021-03-25

**Authors:** Junichi Tsuchiya, Kota Yokoyama, Ken Yamagiwa, Ryosuke Watanabe, Koichiro Kimura, Mitsuhiro Kishino, Chung Chan, Evren Asma, Ukihide Tateishi

**Affiliations:** 1 Department of Diagnostic Radiology and Nuclear Medicine, Tokyo Medical and Dental University, 1-5-45 Yushima, Bunkyo-ku, Tokyo, 113-8510 Japan; 2 Canon Medical Research USA, Inc., 706 N. Deerpath Drive, Vernon Hills, IL 60061 USA

**Keywords:** Deep learning, ^18^F-fluorodeoxyglucose positron emission tomography, Image quality

## Abstract

**Background:**

Deep learning (DL)-based image quality improvement is a novel technique based on convolutional neural networks. The aim of this study was to compare the clinical value of ^18^F-fluorodeoxyglucose positron emission tomography (^18^F-FDG PET) images obtained with the DL method with those obtained using a Gaussian filter.

**Methods:**

Fifty patients with a mean age of 64.4 (range, 19–88) years who underwent ^18^F-FDG PET/CT between April 2019 and May 2019 were included in the study. PET images were obtained with the DL method in addition to conventional images reconstructed with three-dimensional time of flight-ordered subset expectation maximization and filtered with a Gaussian filter as a baseline for comparison. The reconstructed images were reviewed by two nuclear medicine physicians and scored from 1 (poor) to 5 (excellent) for tumor delineation, overall image quality, and image noise. For the semi-quantitative analysis, standardized uptake values in tumors and healthy tissues were compared between images obtained using the DL method and those obtained with a Gaussian filter.

**Results:**

Images acquired using the DL method scored significantly higher for tumor delineation, overall image quality, and image noise compared to baseline (*P* < 0.001). The Fleiss’ kappa value for overall inter-reader agreement was 0.78. The standardized uptake values in tumor obtained by DL were significantly higher than those acquired using a Gaussian filter (*P* < 0.001).

**Conclusions:**

Deep learning method improves the quality of PET images.

## Background

Integrated positron emission tomography (PET) and computed tomography (CT) using ^18^F-fluorodeoxyglucose (FDG) is a standard method used in oncology [1] and is also applied in other conditions, including infectious, ischemic, and degenerative diseases. In oncology, ^18^F-FDG PET/CT is useful for differentiation between benign and malignant lesions, cancer staging, assessment of the response to treatment, and planning of radiation therapy. However, although ^18^F-FDG PET is a promising modality, the images are noisy and their resolution is low [2, 3].

PET images are reconstructed by analytic methods such as filtered back projection [4]. However, reconstructions using analytic methods are challenging because noise statistics related to the emission of photons are difficult to model. Therefore, a statistical model in the maximum likelihood framework has been developed [5].

High-resolution PET images with a high signal-to-noise ratio (SNR) enable better visualization of precise anatomical structures, improving diagnostic accuracy and facilitating early diagnosis of disease and accurate staging. Several methods can be used to obtain high-quality PET images, including increasing the acquisition time, using a time of flight technique, and detection with a semiconductor [6, 7]. In clinical settings, increasing the data acquisition time is a common choice. However, an increased acquisition time leads to a longer examination time, which can be burdensome for patients. Denoising techniques have been used to improve the quality of low SNR images.

A convolutional neural network (CNN) has been applied in medical imaging, including CT and magnetic resonance imaging. Given that it is composed of several linear convolutional layers and nonlinear layers, CNN could reduce statistical noise in PET images without degrading image contrast. Such layers (linear and nonlinear) with a large number of parameters are optimized by training using large datasets. Parameters are optimized by training data extracted from PET/CT datasets. Deep learning approaches for image noise reduction have recently been reported for other modalities, including CT and magnetic resonance imaging [8–11]. Introduction of deep learning-based restoration in single-photon emission tomography (SPECT) images has also been reported. Dietze et al. used a CNN to upgrade images of technetium-99m macroaggregated albumin SPECT/CT pre-treatment, resulting in images comparable with Monte Carlo-based iterative reconstruction, which is known to render better quality images, but to be time-consuming [12]. Moreover, CNN can be applied to PET. CNN was applied for low-dose PET/CT and yielded high-quality images [13–18]. However, the applicability of CNN to clinical PET images has not been fully investigated.

Therefore, the main purpose of this study was to evaluate initial clinical experiences and to explore whether or not the newly developed deep learning-based method improves image quality in comparison with conventional images obtained with a Gaussian filter. We compared whole-body ^18^F-FDG clinical images with the DL method and those with conventional reconstruction both visually and semi-quantitatively.

## Methods

### Patients

The institutional review board of the Tokyo Medical and Dental University approved the present study, and written informed consent was obtained from all patients. The population of the testing studies consisted of 50 patients with a mean age of 64.4 (range, 19–88) years who underwent ^18^F-FDG PET/CT between April 2019 and May 2019. The patient demographic and clinical data are summarized in Table 1.

**Table 1:** Patient demographic and clinical data (*n* = 50)

Age, years	64.9 ± 13.9
Sex	
Male	26
Female	24
Weight, kg	61.9 ± 10.9
Disease, *n*	
Malignancy	
Head and neck tumor	13
Lung cancer	10
Lymphoma	10
Breast cancer	6
Uterine cancer	2
Pancreatic cancer	2
Cancer of unknown primary	2
Others *1	5
Time delay, min	63.7 ± 6.7
Blood sugar level (mg/dl)	115.6 ± 16.0

*1, multiple myeloma, esophageal cancer, colon cancer, malignant melanoma, Takayasu aortitis

### PET/CT imaging

The patients fasted for at least 6 h before undergoing a PET/CT examination and their blood glucose levels were measured. Next, a 3.7-MBq/kg injection of ^18^F-FDG was administered, and ^18^F-FDG imaging was performed approximately 60 min later. Patients were scanned from the skull base to the mid-thigh region using a PET/CT scanner (Celesteion, Canon Medical, Tokyo, Japan). The CT parameters used for attenuation correction were as follows: 120 kV; field of view, 550 mm; pitch, 16.0; and slice thickness, 2.0 mm. PET emission datasets were obtained with 2 min in each bed position (for 16–18 min in total). The conventional PET images with Gaussian postfilter were reconstructed with three-dimensional ordered subset with 2 iterations and 10 subsets. Then, a Gaussian filter of size 6 mm was applied. The DL images were reconstructed with 4 iterations and 10 subsets, and DL-based noise reduction was applied. Next, for semi-quantitative assessment, voxels of interest (VOIs) were calculated. The ^18^F-FDG PET/CT scans were analyzed using a commercially available dedicated Vox-base SP1000 workstation (J-MAC Systems, Sapporo, Japan).

### Deep learning-based approach

Our DL-based approach is an application of deep convolutional neural network (DCNN), which comprises a network training step and a denoising step. In the training step, we prepared a large amount of low-quality and high-quality image pairs in which network parameters are optimized to map a noisy (low-quality) image to a low noise (high-quality) image. The low-quality and high-quality images are also referred to as training images and target images, respectively. The training dataset constituted of 6 patient studies of ^18^F-FDG lung scans with 2 beds coverage (lung to upper abdomen) and 2 × ^18^F-FDG brain studies. The lung scans were acquired for 14 min/bed while the brain studies were scanned for 15 min/bed. These full scans were used as the target images. We then uniformly subsampled the listmode data into 8 noise levels as 30, 45, 60, 120, 180, 240, 300, and 420 s/bed as the noisy training samples. For example, for rebinning a 10-min listmode data into a count level equivalent to a 5-min acquisition, every other event is removed in the listmode data. In such case, these noisy samples share the same clean latent image but are corrupted with different magnitudes of noise. These scan durations cover count levels beyond the normal range seen in clinical practice. In the training process, all the inputs at different noise levels are paired with the same high-quality target [18]. The rationale behind this is that all the noisy samples share the same clean latent image but are corrupted with different levels of noise. The network filters are therefore optimized to estimate the noise residual from different noise levels in the input images. In such a way, the CNN can learn to adapt to different noise levels in the input image automatically and can always produce consistent high-quality images. The training images were reconstructed with an OSEM reconstruction algorithm incorporating time-of-flight (TOF), point spread function (PSF), attenuation, and scatter corrections. All the images were reconstructed with 4 iterations and 10 subsets. The reconstructed image dimension was 272 × 272 × 144 for the lung studies and 120 × 120 × 81 for the brain studies with 2-mm voxel dimension. Before training and testing, all the images were converted to a standardized uptake value (SUV), a quantity that is normalized based on the injected radioactivity and the subject’s body weight. The SUV of normal tissue should be around 1, while a SUV of 2.5 or higher is generally indicative of malignant tissue. All these studies constitute 9234 2D training slices in total. The testing studies are reconstructed using the same protocol and converted to SUV prior to being fed into the network.

### Deep CNN architecture

**Fig. 1:**
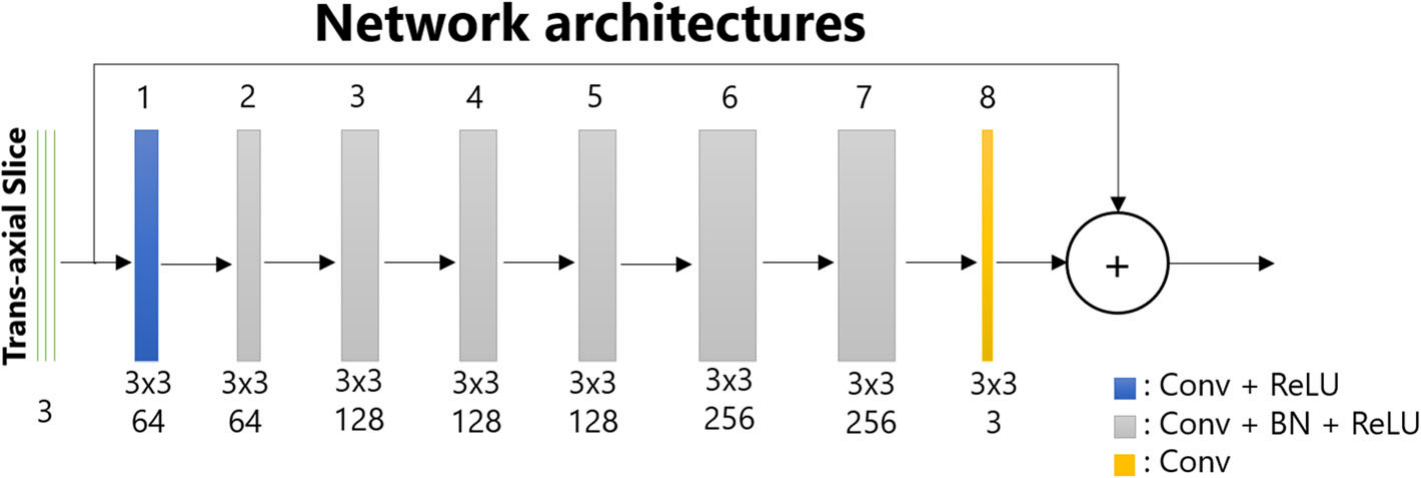
Structure of a deep convolutional neural network. “Conv” represents convolution, “ReLU” denotes a rectified linear unit, and “BN” is an abbreviation for batch normalization

Our DL method consists of a DCNN with 8 layers as shown in Fig. 1. Here, we chose the residual network architecture that tries to estimate the noise **n** instead of high-quality image **x** out of low-quality image **y** = **x** + **n**, as proposed in the previous study [19]. The optimization process then tries to minimize the loss function ***L***:

1 $$ \boldsymbol{L}\left(\boldsymbol{\varTheta} \right)=\frac{1}{N}{\sum}_{i\in N}\psi \left(\mathcal{F}\left({\boldsymbol{y}}_{\boldsymbol{i}};\boldsymbol{\varTheta} \right)-\left({\boldsymbol{y}}_{\boldsymbol{i}}-{\boldsymbol{x}}_{\boldsymbol{i}}\right)\right) $$

where ***Θ*** denotes the trainable weights, $\mathcal{F}\left({\boldsymbol{y}}_{\boldsymbol{i}};\boldsymbol{\varTheta} \right)$ is the estimation of noise **n**, *ψ* is the error function chosen as the mean square error, ***N*** represents the number of training images, ***y*** denotes the training images, and ***x*** denotes the target images.

In conventional DCNN training, the loss function equally weights all voxel-wise differences in image patches. We have proposed a feature-oriented approach in the DCNN training, which uses weight maps to steer the training toward contrast preservation for small features. Phantom and patient studies have demonstrated that this approach can effectively improve contrast recovery on small and low contrast lesions, when the number of high-quality training dataset with small lesions is limited [15].

**Fig. 2:**
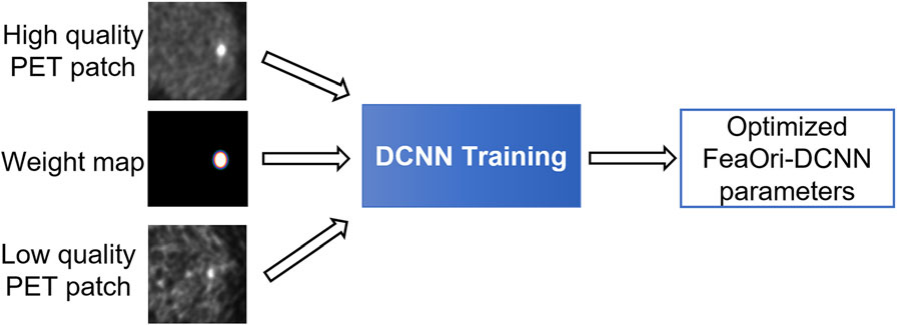
Overview of the feature-oriented deep convolutional neural network. Overview of the feature-oriented deep convolutional neural network training to better preserve small features in output. The weight map is used to assign different weights to different voxels in the loss function calculation. In this case, a higher weight is assigned to voxels in the lesion

**Fig. 3:**
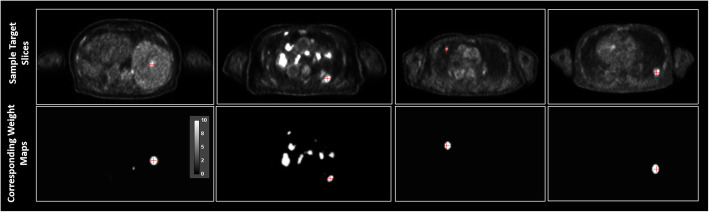
Sample target slices and corresponding weight maps. Trans-axial slices of sample target images and their corresponding weight maps. The red crosshair marks the same location on both slices

As shown in Fig. 2, a weight map that assigns different weights to different voxels is provided in the network along with the low-quality input and high-quality target. The weight map forces the network to learn to preserve the desired small features while suppressing noise in the background. With the weight map, the loss function becomes:

2 $$ \boldsymbol{L}\left(\boldsymbol{\varTheta} \right)=\frac{1}{N}{\sum}_{i\in N}\psi \left(\left(\mathcal{F}\left({\boldsymbol{y}}_{\boldsymbol{i}};\boldsymbol{\varTheta} \right)-\left({\boldsymbol{y}}_{\boldsymbol{i}}-{\boldsymbol{x}}_{\boldsymbol{i}}\right)\right)\bullet {\boldsymbol{w}}_{\boldsymbol{i}}\right) $$

where ***w***_**i**_ denotes the weight map and the ∙ operator denotes voxel-wise multiplication. To generate the weight maps, we first segment the lesions in the target images in the training dataset using adaptive thresholding in order to create the lesion mask (Fig. 3). The weights of the background voxels are set to unity. The voxels in the lesion, which are more important, are set to 10. This value is determined empirically based on the tradeoff between preservation of lesion contrast versus noise reduction in our previous experiments. The weight map is then convolved with a Gaussian kernel to accommodate segmentation errors. The weight map is uniform for the patches that do not contain lesions. The trans-axial slices of sample training targets and the corresponding weight maps are shown in Fig. 3. All the lesions that used to generate the weight maps are located in the lung and liver. Please note the weight map is only needed in the training process that can be casted as a form of regularization in the loss function. Indeed, it prevents the network training overfitting to the noise in the input images. During inference, no weight map is needed.

The inputs and outputs of the networks are 3-channels (3 consecutive slices), which can be considered as a 2.5D convolution process. With stride 1 in the axial direction, some slices may be processed 2 or 3 times. The outputs slices are weight averaged in the axial redirection to form the final volume. During training, patches with a dimension of 32 × 32 × 3 were extracted from the input images with 16, 16, and 2 pixels overlapping in the *x*, *y*, and *z* directions, respectively. During testing, the kernels were applied to the whole image slice. The convolutional kernel dimension was 3 × 3 in order to capture local noise distribution and small features instead of global features. The L2 norm was chosen as the loss function in order to account for high-intensity noise spikes and to obtain stable solution. The Adaptive Moment Estimation (Adam) algorithm optimizer was used to minimize the loss function. The network was trained for 450 total epochs with a gradually decreasing learning rate, i.e., 0.01 for 350 epochs, 0.001 for 50 epochs, and 0.0001 for the last 50 epochs. To validate the training results, we reserved 1% of the training samples for validation during training. These training samples were not used to train the network. Instead, they were used to monitor the performance of the network during training. We computed the loss function using the validation data set and compared to the loss function computed from the training dataset. The network was trained in a Matlab environment using the MatConvNet toolbox [20].

### Qualitative analysis

The acquired images were independently reviewed and analyzed using the Vox-base SP1000 workstation. All PET images were blindly evaluated by two experienced nuclear medicine physicians (with 10 and 8 years of experience, respectively, in interpreting PET scans). The readers were not aware of the clinical indication for PET/CT.

Readers were allowed to manually adjust the standard window settings. Subsequently, based on 5-point scales, the following quality criteria were assessed: tumor delineation (ranging from 1 = lesion cannot be confirmed to 5 = excellent delineation of the lesion margin), overall image quality (ranging from 1 = poor overall image quality to 5 = excellent overall image quality), and image noise (ranging from 1 = enormous image noise to 5 = no perceivable image noise) [21, 22]. In the event of large differences in assessment between readers, the specific images were discussed in a consensus meeting. Readers were instructed to record when they detected any artifact or failure.

### Quantitative analysis

For semi-quantitative analyses, one nuclear medicine physician placed 0.5-mL spherical VOIs in healthy tissues (aortic arch, semioval center [white matter], lung, left ventricle of the heart, parotid gland, quadriceps femoris muscle, and spleen) and also placed a 3-mL spherical VOI in the liver for reference tissue purposes. From these VOIs, the maximum, peak, and mean standardized uptake values (SUVmax, SUVmean, and SUVpeak, respectively) were obtained. VOIs were also placed in the different lesions per patient (with an overall maximum of five lesions per patient, with a maximum of two in the same tissue type). From these measurements, the different SUVmax, SUVpeak, and SUVmean values were compared between the two images. The normalized values of SUV max, SUVpeak, and SUVmean with reference to the aortic arch were calculated and compared between the two images.

### Statistical analysis

Scores for the images acquired using the two methods were compared pairwise using a two-tailed paired samples *t*-test. For inter-reader agreement regarding tumor delineation, overall image quality, and image noise, the original 5-point scores were reassigned to 3-point scores (1 + 2 became 1, 3 became 2, and 4 + 5 became 3). Inter-reader agreement was subsequently evaluated using the kappa statistic. SUV parameters in healthy tissues and lesions were compared between the different systems using a two-tailed paired samples *t*-test. The statistical analysis was performed using SPSS for Windows (IBM Corp., Armonk, NY, USA). A *P*-value < 0.05 was considered statistically significant.

## Results

**Fig. 4:**
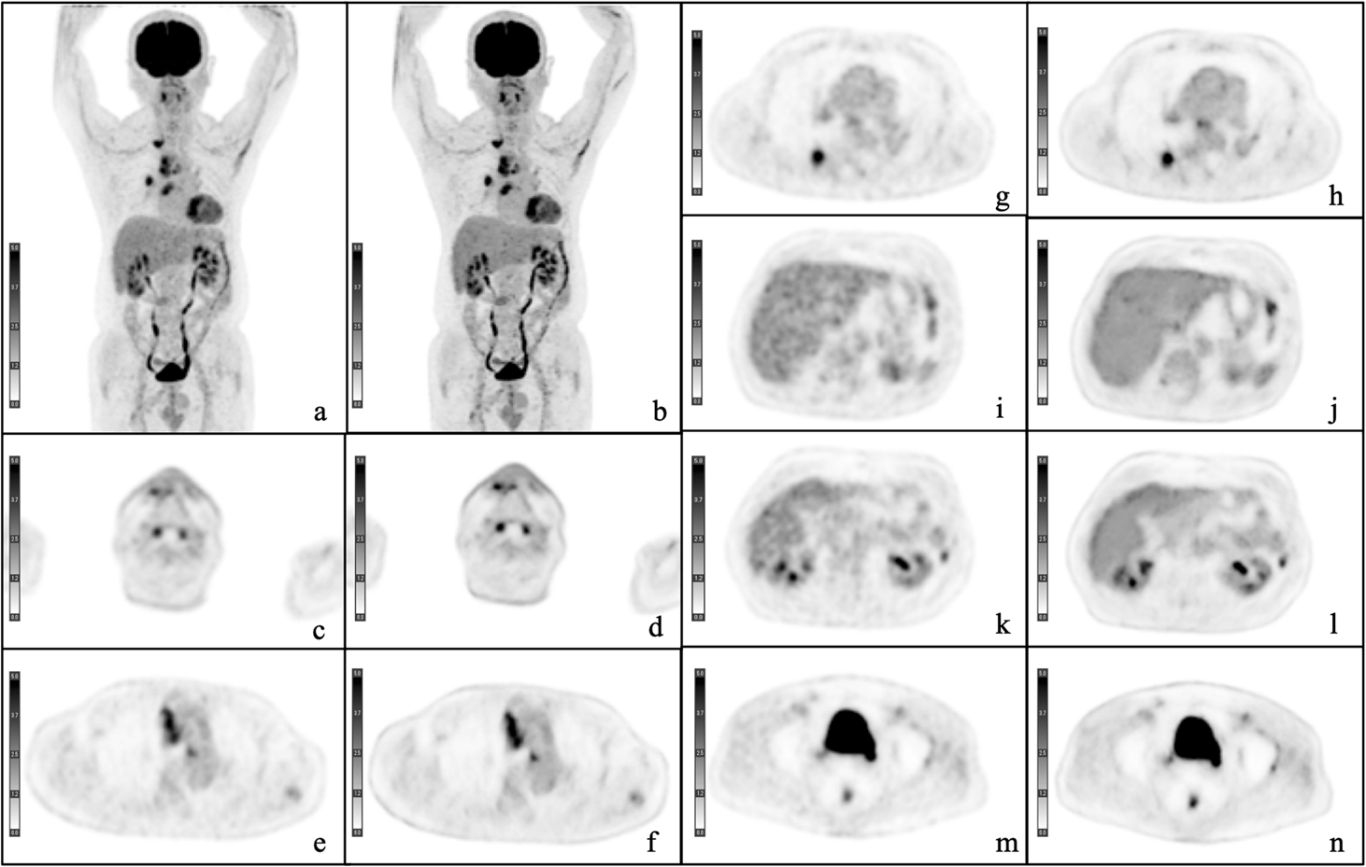
A representative case. A 67-year-old male patient with metastasized non-small cell lung carcinoma. Maximum intensity projection and axial positron emission tomography images with conventional Gaussian filter **a**, **c**, **e**, **g**, **i**, **k**, **m** and those obtained with a deep learning method **b**, **d**, **f**, **h**, **j**, **l**, **n**

**Fig. 5:**
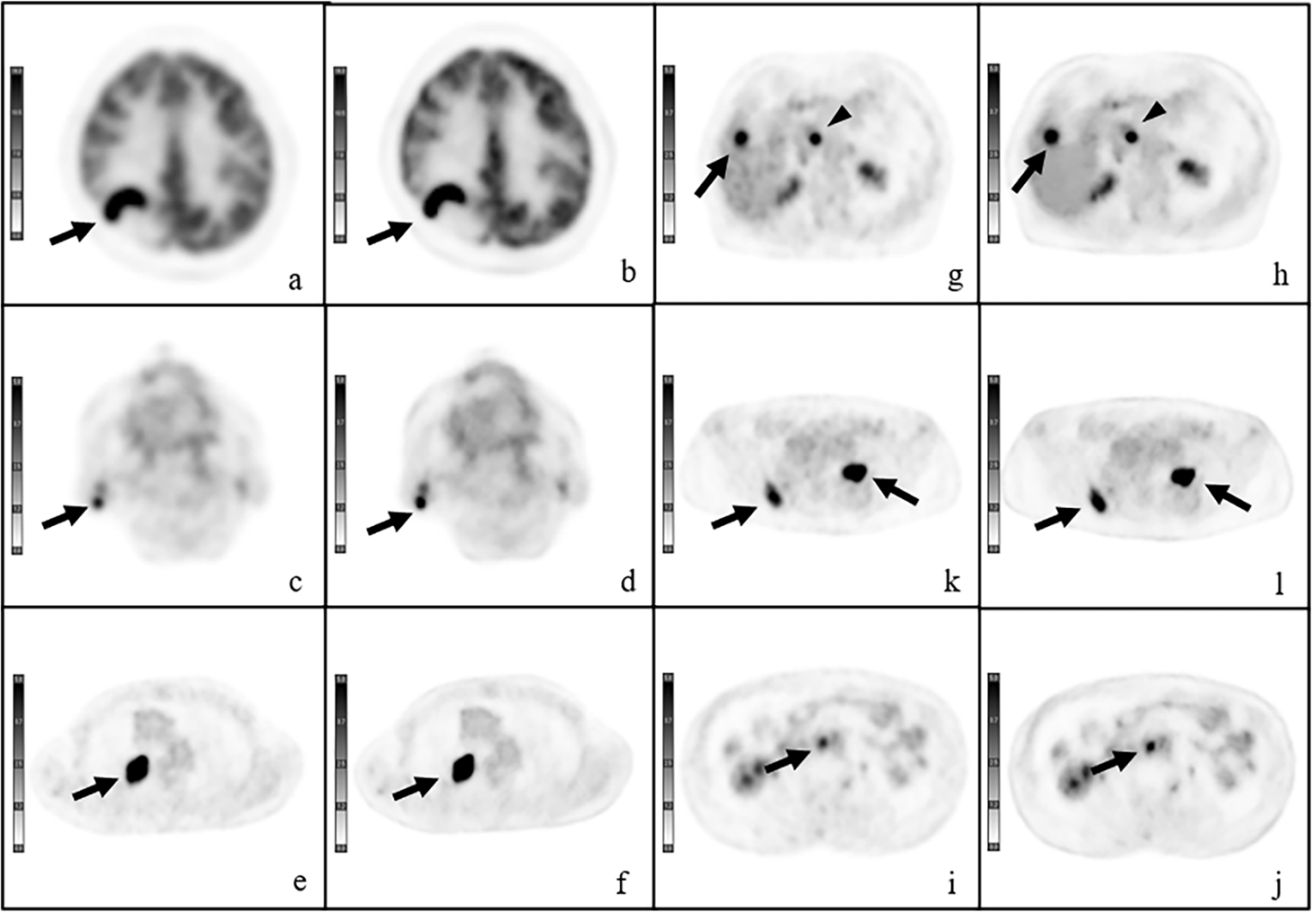
Representative cases. Axial positron emission tomography (PET) images with conventional Gaussian filter **a**, **c**, **e**, **g**, **i** and those obtained with a deep learning method **b**, **d**, **f**, **h**, **l**, **j**. Lymphoma in a 47-year-old woman. Axial PET image shows increased metabolic activity (arrows) in the right parietal lobe **a**, **b**. Warthin tumor in an 87-year-old man with lung cancer. Axial PET image shows increased metabolic activity (arrows) in the right parotid gland **c**, **d**. Recurrent tumor in a 77-year-old man after surgery for lung cancer. Axial PET image shows increased metabolic activity (arrow) in the right lung **e**, **f**. Liver and lymph node metastases in an 88-year-old woman after surgery for lung cancer. Axial PET image shows increased metabolic activity in the right lobe of the liver (arrows) and paraaortic lymph nodes (arrowheads) **g**, **h**. Lymph node metastases in a 70-year-old man after surgery for colon cancer. Axial PET image shows increased metabolic activity (arrow) in the right paraaortic lymph node **i**, **j**

Images using DL were scored significantly higher for tumor delineation, overall image quality, and image noise than at baseline (*P* < 0.001; Table 2). The Fleiss’ kappa value for the overall inter-reader agreement was 0.78. In most of the healthy tissues, the SUVs measured with the DL method were higher than those measured with standard reconstruction (*P* = 0.456 to < 0.001; Table 3). We detected lesions in the region including the brain, parotid gland, thyroid, pharynx, lung, breast, liver, bile duct, pancreas, intestine, lymph nodes (cervical, subclavian, axillary, mediastinum, hepatic, paraaortic, and inguinal), subcutaneous area, and bones (rib, spine, and pelvic bones). The sizes of the lesions in the maximal axial plain ranged from 0.52 to 102.21 cm^3^. The difference between DL images and Gaussian filtered images was more significant in tumor tissues (*P* = 0.31 to < 0.001; Table 4). The difference in normalized values between DL images and Gaussian filtered images was also significant (*P* = 0.09 to < 0.001; Table 5). Representative cases are shown in Figs. 4 and 5. No artifact or failure was detected.

**Table 2:** Qualitative image analysis

	dPET	cPET	***P***-value
**Delineation**	4.06 ± 0.24	2.94 ± 0.24	< 0.0001
**Noise**	3.88 ± 0.48	2.40 ± 0.50	< 0.0001
**Overall image quality**	3.94 ± 0.44	2.98 ± 0.20	< 0.0001

The data are shown as the mean and standard deviation. *cPET*, conventional positron emission tomography; *dPET*, deep learning processed positron emission tomography

**Table 3:** SUVs in healthy organ tissues

Organs		cPET mean ± SD	dPET mean ± SD	*P*-value
Aortic arch	SUV max	1.63 ± 0.26	1.72 ± 0.26	< 0.0001
	SUV mean	1.34 ± 0.20	1.44 ± 0.20	< 0.0001
	SUV peak	1.48 ± 0.21	1.57 ± 0.21	< 0.0001
Semioval center	SUV max	2.28 ± 0.43	2.19 ± 0.39	0.04
	SUV mean	1.85 ± 0.45	1.76 ± 0.37	0.003
	SUV peak	2.28 ± 0.43	2.37 ± 0.47	< 0.0001
Liver	SUV max	2.25 ± 0.43	2.09 ± 0.27	< 0.0001
	SUV mean	1.76 ± 0.33	1.81 ± 0.30	0.456
	SUV peak	2.00 ± 0.37	1.92 ± 0.24	< 0.0001
Lung	SUV max	0.33 ± 0.30	0.31 ± 0.09	0.033
	SUV mean	0.26 ± 0.23	0.24 ± 0.07	< 0.0001
	SUV peak	0.31 ± 0.27	0.29 ± 0.08	0.006
Left ventricle	SUV max	1.62 ± 0.29	1.64 ± 0.27	0.19
	SUV mean	1.32 ± 0.24	1.41 ± 0.27	< 0.0001
	SUV peak	1.59 ± 0.32	1.63 ± 0.30	0.006
Parotid gland	SUV max	1.33 ± 0.37	1.49 ± 0.43	< 0.0001
	SUV mean	1.11 ± 0.30	1.20 ± 0.33	< 0.0001
	SUV peak	1.22 ± 0.34	1.30 ± 0.37	< 0.0001
Quadriceps muscle	SUV max	0.64 ± 0.16	0.67 ± 0.17	< 0.0001
	SUV mean	0.50 ± 0.12	0.54 ± 0.12	< 0.0001
	SUV peak	0.58 ± 0.13	0.60 ± 0.13	< 0.0001
Spleen	SUV max	1.87 ± 0.30	1.92 ± 0.31	0.023
	SUV mean	1.59 ± 0.26	1.30 ± 0.37	< 0.0001
	SUV peak	1.70 ± 0.26	1.69 ± 0.24	< 0.0001

The data are shown as the mean and standard deviation. Note: The current DCNN is trained to be used only for general whole-body studies but is not designed for the brain. *cPET*, conventional positron emission tomography; *dPET*, deep learning processed positron emission tomography; *SD*, standard deviation; *SUV*, standardized uptake value

**Table 4:** SUVs in tumor lesions (*n* = 108)

		cPET mean ± SD	dPET mean ± SD	*P*-value
Lesions	SUV max	5.59 ± 3.97	8.42 ± 5.02	< 0.0001
	SUV mean	1.98 ± 0.96	2.14 ± 0.83	< 0.0001
	SUV peak	4.04 ± 2.93	4.69 ± 3.07	0.31

The data are shown as the mean and standard deviation. *cPET*, conventional positron emission tomography; *dPET*, deep learning processed positron emission tomography; *SD*, standard deviation

**Table 5:** Difference in SUV ratios compared to the background SUV in tumor lesions (*n* = 108)

		cPET Mean ± SD	dPET Mean ± SD	*P*-value
Lesions	SUV max ratio	3.42 ± 2.32	4.95 ± 3.26	< 0.0001
	SUV mean ratio	1.49 ± 0.73	1.52 ± 0.69	0.09
	SUV peak ratio	2.81 ± 2.05	3.09 ± 2.26	< 0.0001

The data are shown as the mean and standard deviation. *cPET*, conventional positron emission tomography; *dPET*, deep learning processed positron emission tomography; *SD*, standard deviation

## Discussion

This study evaluated the effect of the deep learning technique on the quality of FDG PET/CT images. In DL images, the image noise was lower, and the lesion delineation and image quality were superior compared to conventional reconstruction. DL enhanced image quality and reduced image noise.

A conventional spatial filter, such as the Gaussian filter, involves mixing of pixel values and the amount or ratio of mixture is determined simply by the distance between two pixels. This process results in unwanted blurring of organs and reduction of the SUV value of regions with small concentration in both tumor and healthy organs. The principle underlying the deep learning noise reduction process is different from the conventional convolution filter approach. When noisy data are used as the training input and high-quality data are presented as the training target, the network can learn to produce clean images from noise-contaminated images. Our training target dataset for DCNN comprised high-quality images with a longer acquisition time. DL imaging rendered a higher image quality than conventional imaging with Gaussian filtering. The difference between the conventional filter approach and DL approach results in a difference in the SUV parameters. For tumors and healthy tissues in small organs, Gaussian filtering shaves off pixel values in the images, while DL only removes noise. This difference results in a higher SUVmean in most concentrations. When we consider relatively larger organs like the liver, excluding its edge, both Gaussian filtering and DL do not change the overall SUV level. Thus, the lower SUVmax and SUVpeak of DL demonstrate that DL has stronger denoising capability than Gaussian filtering.

In the qualitative assessment, we compared DL PET images with conventional PET images. DL reduced the image noise and improved the image quality. Schaefferkoetter et al. demonstrated that the denoising method using CNN improved very noisy data. However, the lesser effect was appreciated by applying the technique to routinely encountered images [17]. The clinical impact of the DL method has to be investigated in future studies. Moreover, the application of the DL technique used in the current study may lead to a reduction in the radiation dose and a shorter acquisition time. Cheng et al. enhanced the image quality of 2 one-hundredth dose PET to that of standard-dose PET using the deep learning method [13]. Further studies are necessary to determine whether or not the diagnostic accuracy of imaging with DL methods used in this study is retained at reduced radiation doses.

PET images have a low SNR so need denoising. The commonly used technique is Gaussian filtering, which is sometimes implemented in iterative image reconstruction algorithms [23]. Gaussian filtering can increase the SNR. However, it also smooths the image and can produce a loss of resolution by averaging voxels together and blurring the distinction between two closely adjacent objects.

The network used in this study was specifically trained and applied on ^18^F-FDG PET studies acquired on Canon Medical’s Celesteion PET/CT scanner. The application of this network to other imaging tracers or scanners may not produce equivalent results as presented in this study, which requires further investigation. In addition, we have not investigated the impact of dose/scan duration reduction on the diagnostic quality of the network results, which also warrants future investigation.

## Conclusions

PET imaging is already characterized by limited spatial resolution and associated partial volume effects. The DL methods used in this study enhanced the image quality by maintaining the values of the SUV parameters. In this regard, the DL technique is superior to conventional reconstruction.

In this initial study, we found that the DL method provides better perceived image quality than conventional imaging with a Gaussian filter; more sharply demarcated tumor lesions were seen, the overall image quality was higher, and a higher SNR was assessed visually. In terms of semi-quantitative image quality, the DL technique renders higher values for SUV parameters on imaging of tumors and healthy tissues in small organs, while Gaussian filtering decreases the SUV by blurring. Our results demonstrate that DL respects the tissue boundaries well and reduces the noise considerably without losing quantitative information on PET images, including SUVmax and SUVmean, when compared with images using a Gaussian filter. However, improved quantitative performance may be feasible using clinically optimized reconstruction settings. Future studies that include groups of patients with more homogeneous oncologic disease are necessary to validate our findings and to assess the potential clinical impact of the DL method on PET imaging.

## Data Availability

The datasets generated during and/or analyzed during the current study are available from the corresponding author on reasonable request.

## Abbreviations

CNN: Convolutional neural network
CT: Computed tomography
DL: Deep learning
DCNN: Deep convolutional neural network
FDG: Fluorodeoxyglucose
PET: Positron emission tomography
SNR: Signal-to-noise ratio
SPECT: Single-photon emission tomography
SUV: Standardized uptake value
VOI: Voxel of interest
